# Big data analysis of the risk factors and rates of perioperative transfusion in immediate autologous breast reconstruction

**DOI:** 10.1038/s41598-022-09224-7

**Published:** 2022-03-29

**Authors:** Woo Jin Song, Hee Jin Kim, Sang Gue Kang, Bommie Florence Seo, Nam Kyong Choi, Jung Ho Lee

**Affiliations:** 1 Department of Plastic and Reconstructive Surgery, Soonchunhyang University College of Medicine, Seoul, Republic of Korea; 2 Department of Plastic and Reconstructive Surgery, Uijeongbu St. Mary’s Hospital, College of Medicine, The Catholic University of Korea, Uijeongbu, Republic of Korea; 3 Department of Plastic and Reconstructive Surgery, Bucheon St. Mary’s Hospital, College of Medicine, The Catholic University of Korea, 327, Sosa-ro, Bucheon-si, Gyeonggi-do 14647 Republic of Korea; 4 Department of Health Convergence, Ewha Womans University, 52 Ewhayeodae-gil, Seodaemungu, Seoul, 03760 Republic of Korea; 5 Korean Academic Association of Aesthetic and Reconstructive Breast Surgery, Seoul, Republic of Korea

**Keywords:** Medical research, Oncology, Risk factors

## Abstract

Patients undergoing autologous breast reconstruction (ABR) are more likely to require perioperative transfusions due to the increased intraoperative bleeding. In addition to the mastectomy site, further incisions and muscle dissection are performed at the donor sites, including the back or abdomen, increasing the possibility of transfusion. The purpose of this study was to evaluate perioperative transfusion rates and risk factors according to the type of ABR through analysis of big data. Patients who underwent total mastectomy for breast cancer between 2014 and 2019 were identified. The patients were divided into mastectomy only and immediate ABR groups. The transfusion rate was 14-fold higher in the immediate ABR group (16.1%) compared to the mastectomy only group (1.2%). The transfusion rate was highest with the pedicled transverse rectus abdominis myocutaneous flap (24.2%). Performance of the operation in medical institutions located in the provinces and coronary artery disease (CAD) were significant risk factors for the need for transfusion. The perioperative transfusion risk among patients undergoing immediate ABR was related to the flap type, location of medical institution, and CAD. Based on the higher transfusion rate in this study (16.1%) compared to previous studies, the risk factors for the need for transfusion should be determined and evidence-based guidelines should be developed to reduce the transfusion rates.

## Introduction

Implant breast reconstruction (IBR) is a recently introduced technique associated with high risks of capsular contracture and reconstruction failure in patients who receive radiation therapy. In high-risk patients, the only option available is autologous breast reconstruction (ABR)^1^. The average reoperation rate after ABR is 1.06 times, which is lower than that after IBR. The higher reoperation rate after IBR is due to implant-related complications, such as capsular contracture and implant malposition^2,3^. In addition, ABR is associated with improved quality of life, high satisfaction rates, and long-lasting results. ABR creates natural-looking contours that are almost identical to the natural ones.

However, ABR is associated with a longer operative time, high risk of intraoperative bleeding and need for perioperative transfusion^4^. Transfusion rates of 8.2–80.3% for the deep inferior epigastric artery perforator (DIEP) flap and 1.6–95% for the transverse rectus abdominis myocutaneous (TRAM) flap have been reported during ABR^5–13^. This wide variation in transfusion rates is probably due to transfusions being based on the individual decisions of the surgeon or institutional policy. The leading perioperative morbidities of blood transfusion are allergic reaction, bacterial infection, viral infection (HIV, HBV, and HCV), acute lung injury, anaphylactic shock, graft-versus-host disease, and immunosuppression, which result in increased mortality, prolonged hospitalization, and increased healthcare costs^14^. Therefore, the risk factors for the need for perioperative transfusion need to be determined to minimize transfusion rates.

There are scarce data comparing transfusion rates between the different flap types used during ABR. To our knowledge, no studies have analyzed transfusion rates and the risk factors for the need for transfusion during ABR according to flap type. In this study, we evaluated the risk factors for ABR perioperative transfusion, and the rates thereof, according to flap type, using the data stored in the Big Data Hub of the Health Insurance Review and Assessment Service (HIRA).

## Methods

### Study populations and data source

We collected data from the HIRA database, which contains information on claims for prescribed medications, diagnoses, surgical procedures, prescription records, and demographic information for approximately 50 million Koreans. Patients in the HIRA database can be identified by their unique Korean Resident Registration Number, assigned to each Korean resident at birth, which prevents duplications or omissions during data collection^15^. All data included in this study were anonymized.

The study population consisted of women aged 18–59 years who underwent total mastectomy for breast cancer between January 1, 2014 and May 31, 2019. To identify the newly diagnosed patients, we included all women assigned the V193 code (International Statistical Classification of Disease and Related Health Problems, 10^th^ Revision, ICD-10 code C50 or D05) between April 1, 2015 and December 31, 2017, and excluded patients diagnosed with breast cancer between April 1, 2014 and March 31, 2015. The V193 code is used for patients diagnosed with breast cancer whose out-of-pocket medical expenses for cancer treatment are reduced to 5% of the total cost by the National Health Insurance Service (NHIS)^16^. We used the Healthcare Common Procedure Coding System of the HIRA to identify the procedure or surgery (Supplementary Table S1). Patients who underwent total mastectomy were identified based on the relevant codes (N7130, N7135, N7138, or N7139). We excluded women who underwent IBR (N7148, N7149, N7150, or N7151). To determine the transfusion volume for each surgery, we excluded patients who underwent bilateral mastectomy. The claims data did not include information on bilateral surgeries; therefore, we excluded patients assigned two or more codes for total mastectomy on the day of surgery. We also excluded patients who underwent ABR before total mastectomy because they may have been previously diagnosed. We divided the study participants into mastectomy only and mastectomy with immediate ABR groups. Patients who were assigned codes for both total mastectomy and autologous transplant (N7140, N7141, N7142, N7143, N7144, N7145, N7146, or N7147) were included in the immediate ABR group.

### Transfusion

We identified patients who received a transfusion based on the codes assigned on the day of surgery (X2021 and X2022 for 320 and 400 mL infusions, respectively). The number of units was also calculated based on the codes. For example, for a patient assigned one X2021 and one X2022 code, the transfusion volume was recorded as 720 mL of whole blood and red blood cells (two units).

### Statistical analyses

We compared the demographic characteristics between the mastectomy only and immediate ABR groups, including age, medical institution region, type of medical institution, and insurance type. The two medical institution region categories were provinces and cities (including Seoul, Incheon, Daejeon, Gwangju, Daegu, Ulsan, and Busan). In addition, medical institutions were classified as tertiary (including university hospitals and general hospitals), secondary (including hospitals and nursing hospitals), or primary (including clinics and public health centers) institutions. Health insurance was divided into National Health Insurance and Medical Aid. About 97% of Koreans are covered by National Health Insurance, while the remaining 3% are covered by Medical Aid, including the low-income beneficiaries of the Korean National Basic Livelihood Security System^17^. Hypertension, diabetes mellitus (DM), coronary artery disease (CAD), chronic obstructive pulmonary disease (COPD), end-stage renal disease (ESRD), anemia, and liver disease were recorded based on the codes assigned in the year preceding the date of surgery. Descriptive statistics were used to compare the rates and volumes of transfusion between the two groups. The rates and volumes of transfusion for ABR patients were compared between different flap types. The flaps included latissimus dorsi myocutaneous (LD), pedicled TRAM (pTRAM), free TRAM (fTRAM), and DIEP flaps. We estimated the odds ratios (ORs) and 95% confidence intervals (CIs) for transfusion for each surgical method using a multiple logistic regression model that included age, region, insurance type, medical institution type, and comorbidities (including hypertension, DM, CAD, COPD, ESRD, anemia, and liver disease). Multiple logistic regression was used to identify the risk factors for transfusion in immediate ABR patients. A *p* value < 0.05 was used to determine statistical significance. All analyses were performed using SAS statistical software (version 9.4; SAS Institute, Cary, NC, USA).

### Waiver of consent

Informed consent is not obtained from subjects. The research could not practicably carry out without a waiver. Identifying and contacting the more than 30,000 of potential subjects, although not impossible, would not be feasible for a review of their medical records for information that would not change the care they would already have received. The institutional review board of Bucheon St. Mary Hospital (IRB No. HC2020-0300-0001) approved a waiver of informed consent.

### Ethical approval

This study was approved by the institutional review board of Bucheon St. Mary Hospital (IRB No. HC2020-0300-0001) and performed in accordance with the Declaration of Helsinki.

## Results

**Figure 1:**
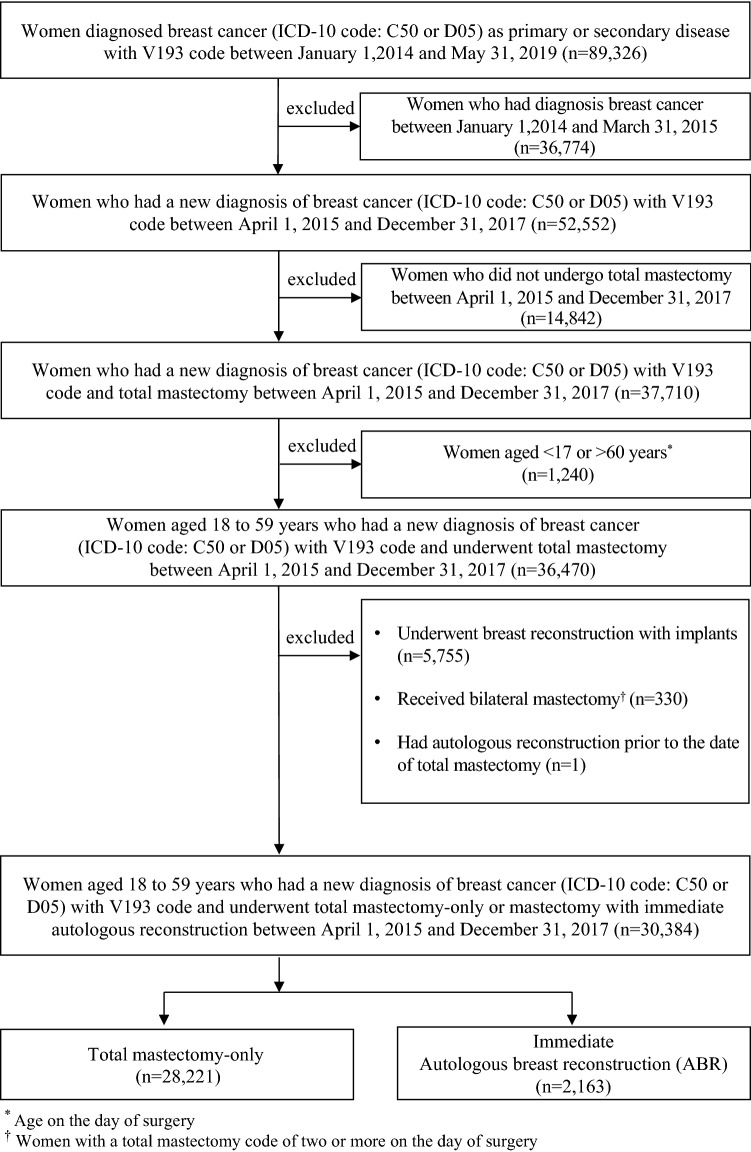
Flowchart of the selection of the study population.

Of the 30,384 patients included in this study, 28,221 underwent total mastectomy only and 2163 underwent total mastectomy with immediate ABR (Fig. 1). Middle-aged women were in the majority in both groups. Most patients in the mastectomy only and immediate ABR groups underwent surgery at tertiary medical institutions (97.5% and 98.8%, respectively) located in cities (70% and 75.6%, respectively), and were covered by the National Health Insurance (97.5% and 99.1%, respectively). In the mastectomy only and immediate ABR groups, COPD was the most common comorbidity (23.2% and 23.6%, respectively), followed by liver disease (15.6% and 15.0%, respectively), and hypertension (14.7% and 12.8%, respectively) (Table 1). The overall transfusion rates for the mastectomy only and immediate ABR groups were 1.2% and 16.1%, respectively. The transfusion rates for the immediate ABR group were approximately 14 times higher than for the mastectomy only group. The transfusion volume was typically 1 or 2 units regardless of whether immediate ABR was performed. However, the maximum transfusion volume differed between the mastectomy only and the immediate ABR groups, 720 and 1840 mL, respectively (*p* < 0.001) (Table 2).

**Table 1:** Baseline characteristics of the study population.

	Total mastectomy only N (%)	Immediate ABR N (%)
**Total**	28,221 (100.0)	2163 (100.0)
**Age group (y)**		
18–29	259 (0.9)	18 (0.8)
30–39	2986 (10.6)	306 (14.2)
40–49	12,370 (43.8)	1136 (52.5)
50–59	12,606 (44.7)	703 (32.5)
**Region**		
City*	19,750 (70.0)	1635 (75.6)
Province	8471 (30.0)	528 (24.4)
**Insurance type**		
National health insurance	27,512 (97.5)	2143 (99.1)
Medical aid	709 (2.5)	20 (0.9)
**Type of medical institution**		
Tertiary	27,511 (97.5)	2137 (98.8)
Secondary	449 (1.6)	22 (1.0)
Primary	261 (0.9)	4 (0.2)
**Comorbidities**		
Hypertension	4139 (14.7)	277 (12.8)
Diabetes	2655 (9.4)	173 (8.0)
Coronary artery disease	657 (2.3)	42 (1.9)
Chronic obstructive Pulmonary disease	6,550 (23.2)	511 (23.6)
End stage renal disease	54 (0.2)	2 (0.1)
Anemia	6 (0.0)	0 (0.0)
Liver disease	4404 (15.6)	325 (15.0)

*Seoul, Busan, Incheon, Daegu, Ulsan, Gwangju, and Daejeon. *ABR* autologous breast reconstruction.

**Table 2:** Transfusion rates and volumes for the entire study population.

	Total mastectomy only N (%)	Immediate ABR N (%)	*P*
**Total**	28,221 (100.0)	2163 (100.0)	
**Transfusion, n (%)**			< 0.001
Yes	350 (1.2)	349 (16.1)	
No	27,871 (98.8)	1814 (83.9)	
**Transfusion volume (mL)**			< 0.001
Average (± SD)	433.1 (± 143.0)	517.4 (± 273.5)	
Median	400	400	
Max, Min	720, 320	1840, 320	
**Transfusion volume (units), n (%)**			< 0.001
1–2	350 (100.0)	321 (92.0)	
3–4	–	25 (7.2)	
≥ 5	–	3 (0.9)	

*ABR* autologous breast reconstruction, *SD* standard deviation, *Max* maximum, *Min* minimum.

In the immediate ABR group, 36.5% of patients received DIEP, 21.3% received fTRAM, 19.3% received LD, and 16.8% received pTRAM flaps (Table 3). The transfusion rates were highest in the pTRAM patients (24.2%), followed by the fTRAM (21.3%), DIEP (11.9%), and LD (9.8%) patients. The relative risks for transfusion were significantly higher for pTRAM (OR = 3.06; 95% CI = 2.03–4.63; *p* < 0.001) and fTRAM (OR = 2.50; 95% CI = 1.68–3.71; *p* < 0.001), but not for DIEP, compared to LD. The average transfusion volume was highest with DIEP (538.7 ± 335.2 mL), followed by fTRAM (530.8 ± 268.4 mL), pTRAM (519.1 ± 251.8 mL), and LD (423.4 ± 123.5 mL) flaps. Among the immediate ABR patients, the risk factors for the need for transfusion were medical institution located in the provinces (OR = 1.47; 95% CI = 1.13–1.90; *p* = 0.004) and CAD (OR = 2.11; 95% CI = 1.06–4.21; *p* = 0.03) (Table 4).

**Table 3:** Transfusion rates, volumes, and relative risks according to the type of surgical method in immediate autologous breast reconstruction patients.

Autologous type	N (%)	Transfusion rate, n (%)	Average transfusion volume (mL)	Average transfusion volume (unit)	Adjusted OR* (95% CI)	*P*
LD flap	418 (19.3)	41 (9.8)	423.4 (± 123.5)	1.0	1.00 (Reference)	
Pedicled TRAM	363 (16.8)	88 (24.2)	519.1 (± 251.8)	1.1	3.06 (2.03–4.63)	< .001
Free TRAM	592 (27.4)	126 (21.3)	530.8 (± 268.4)	1.1	2.50 (1.68–3.71)	< .001
DIEP	790 (36.5)	94 (11.9)	538.7 (± 335.2)	1.1	1.32 (0.88–1.97)	.18

*OR* odds ratio, *LD* latissimus dorsi, *TRAM* transverse rectus abdominis, *DIEP* deep inferior epigastric perforator, *CI* confidence interval.

*Estimated by logistic regression adjusted for age, region, insurance type, medical institution type, and comorbidities (including hypertension, diabetes mellites, coronary artery disease, chronic obstructive pulmonary disease, end-stage renal disease, anemia, and liver disease).

**Table 4:** Risk factors for transfusion in immediate autologous breast reconstruction patients.

	Transfusion N	Non-transfusion N	Adjusted OR* (95% CI)	*P*
**Total**	349	1814		
**Age (y)**				
18–29	3	15	1.00 (Reference)	
30–39	42	264	0.75 (0.21–2.73)	.67
40–49	199	937	0.97 (0.28–3.41)	.97
50–59	105	598	0.78 (0.22–2.78)	.71
**Region**				
City^†^	242	1,393	1.00 (Reference)	
Province	107	421	1.47 (1.13–1.90)	.004
**Insurance type**				
National health insurance	343	1,800	1.00 (Reference)	
Medical aid	6	14	2.51 (0.95–6.66)	.06
**Type of medical institution**				
Tertiary	1	3	1.00 (Reference)	
Secondary	2	20	0.56 (0.13–2.39)	.43
Primary	346	1,791	1.27 (0.13–12.38)	.84
**Comorbidities**				
Hypertension	47	230	1.07 (0.74–1.54)	.72
Diabetes	30	143	1.12 (0.74–1.54)	.62
Coronary artery disease	12	30	2.11 (1.06–4.21)	.03
Chronic obstructive pulmonary disease	69	442	0.76 (0.57–1.01)	.06
End stage renal disease	1	1	5.20 (0.32–83.62)	.25
Anemia	–	–	–	–
Liver disease	53	296	1.06 (0.76–1.48)	.73

*OR* odds ratio, *CI* confidence interval.

*Estimated by logistic regression adjusted for age, region, insurance type, medical institution type, and comorbidities (including hypertension, diabetes mellites, coronary artery disease, chronic obstructive pulmonary disease, end-stage renal disease, anemia, and liver disease).

^†^Seoul, Busan, Incheon, Daegu, Ulsan, Gwangju, and Daejeon.

## Discussion

In this retrospective study, we performed a detailed analysis of big data to determine the rates and risk factors for perioperative transfusion by ABR type. The hemoglobin level determines the oxygen concentration of the arterial blood, and an adequate hemoglobin level is essential for flap success after ABR^18^. The preoperative hemoglobin level and surgical blood loss are used to determine whether a transfusion is required^9^. Adequate hemoglobin levels for wound healing or flap survival are difficult to establish due to comorbidities that vary from patient to patient. However, the intraoperative hemoglobin level may be a guideline for transfusions. Blood transfusions are necessary in cases of severe blood loss during surgery. However, blood transfusions may pose risks due to problems with the storage process, cytokine release, and inflammatory response. Transfusion-associated circulatory overload, acute lung injury, immunological reactions, infections, venous thromboembolism, and fat necrosis may complicate blood transfusions and lead to increased mortality, duration of hospitalization, and medical costs, as well as reconstruction failure^13,19^.

Although the exact mechanism is unclear, it is believed that lymphocyte dysfunction caused by blood transfusions affects tumor recurrence and the survival rate. Allogenic blood transfusion is known to downregulate macrophages and T cell immunity and suppresses the immune system by increasing glucocorticoid levels^20–22^. In a propensity score analysis of 4030 patients who underwent resection for stage I–III colorectal cancer, blood transfusion was associated with shorter disease-free and overall survival rates. Additionally, larger transfusion volumes were associated with higher overall mortality^23^. Previous studies have reported conflicting results regarding the effects of blood transfusion in breast cancer patients. A meta-analysis performed in 2007 reported that transfusion did not correlate with the survival of breast cancer patients. However, a study conducted in 2018 reported that perioperative transfusions negatively impacted the survival of breast cancer patients^24,25^. Therefore, the risk factors for the need for transfusion in patients undergoing surgery for breast cancer should be identified. According to our study, the risk of transfusion in breast cancer patients who underwent immediate ABR increased when they had surgery at a provincial medical institution or had CAD.

Previous studies have reported a wide range of transfusion rates in patients undergoing ABR. This variability is a result of a lack of well-defined criteria for blood transfusion. Most patients in previous studies received transfusions based on the preference of surgeons or anesthesiologists. In one study, Holley et al. reported a 95% transfusion rate in 50 ABR patients (including pTRAM, fTRAM, and LD flaps) with a mean volume of 2.4 units per patient^10^. In a meta-analysis by Rinker et al., the overall transfusion rate was 47% for ABR patients, 30% for the fTRAM flap, and 56% for the pTRAM flap^24^. According to a recent US nationwide inpatient sample database study, the overall rate of blood transfusion after ABR was 7.0%, and the risk was increased by chronic anemia, congestive heart failure, use of a free flap, chronic renal failure, hypertension, chronic lung disease, diabetes, obesity, and operation performance at a non-teaching hospital^26^.

In this study, the need for transfusion was greater for medical institutions located in the provinces, and in the presence of CAD. A high risk of transfusion in CAD patients has been reported in several studies. CAD patients show impairment in the compensatory mechanisms required to maintain oxygenation in the presence of anemia; anemia is associated with worse outcomes, more severe illness, and mortality in CAD patients^27^. Docherty et al. suggested a more liberal transfusion threshold (> 8.0 g/dL) for patients with cardiovascular disease^28^. CAD patients also require perioperative aspirin to prevent cardiovascular complications. Among patients undergoing coronary artery bypass grafting or valve replacement, the number receiving red blood cell transfusions was higher in the aspirin-using group than in the controls^29^. In our study, patient age, diabetes, and previous anemia were not significant risk factors for blood transfusion.

The transfusion rate was highest for the pTRAM flap (32%), followed by the fTRAM (27%), DIEP (13.5%), and LD (10.9%) flaps. The transfusion rates for the pTRAM and fTRAM flaps were significantly higher than for the LD flap. The transfusion rates were higher for pTRAM than fTRAM, similar to the findings of Rinker et al.^24^ Drazan et al. reported that 7.2% of patients received a transfusion during bilateral breast reconstruction with DIEP flaps, and the mean blood transfusion volume was 810 mL^7^. Appleton et al. reported that 18.8% of patients with a DIEP flap received a transfusion, with an average transfusion volume of 1170 mL^11^. In this study, the average transfusion volume was highest for the DIEP flap (538.7 ± 335.2 mL), followed by the fTRAM (530.8 ± 268.4 mL), pTRAM (519.1 ± 251.8 mL), and LD (423.4 ± 123.5) flaps.

In a literature review, the transfusion rate was between 1.6 and 95%, and this difference was reported to be related to the breast reconstruction method. The transfusion rate is affected by the complexity and frequency of the procedure performed according to the ABR type, and this is thought to be why the transfusion rate of LD flaps is lower than that of other flaps^26,30^. The transfusion rate of fTRAM or DIEP flaps was reported to be higher or no different than that of pTRAM flaps, and overall complications such as hernia, hematoma or infection were reported to be higher with pTRAM, which was similar to our result^30,31^. Other studies have reported that the ABR type does not significantly affect the transfusion rate, although a large comparative study of this is needed. Nevertheless, low preoperative hemoglobin levels and bilateral DIEP flaps were two major risk factors increasing the transfusion rate^26^. TRAM flaps require more muscle dissection and increase the surgical blood loss, while with the DIEP flap, longer operating times and increased flap weight are associated with increased transfusion volume^32^. Positive-balance fluid management after fTRAM flap is also associated with increased transfusion volume by causing hemodilution and relative anemia^33^.

In this study, the overall rate of blood transfusion was 16.1%, which was higher than in a US study. The transfusion rate was significantly higher in our immediate ABR group compared to the mastectomy only group. These results suggest that efforts are needed to reduce transfusion rates in patients undergoing ABR. Special efforts should be made to reduce the need for transfusion in high-risk patients by minimizing blood loss and using cell savers. Previous studies have shown that the tumescent technique with diluted local anesthesia and epinephrine before the incision during TRAM flap harvesting reduced perioperative bleeding. The transfusion rate is also reduced by electrocautery during mastectomy and flap elevation^12,34^. Preoperative anemia increases the risk of transfusion with the DIEP flap and the risk can be reduced by controlling the reversible risk factors preoperatively (e.g., via iron supplementation)^26^. In addition, more restrictive transfusion criteria are necessary in patients undergoing ABR. It is still difficult to obtain consensus on restricted versus liberal blood transfusion. To prevent free flap failure, some reports recommend liberal transfusion when the hemoglobin is less than 8.75 or 10 g/dL^18,35^. However, the updated blood transfusion guidelines suggest a transfusion threshold of 7 g/dL for hemodynamically stable patients, including critically ill patients. Therefore, a restrictive transfusion strategy should be used, with transfusion done only if the hemoglobin is less than 7 g/dL or the patient is symptomatic. This strategy leads to fewer transfusion episodes and no increase in microvascular complications^13,36,37^.

There were several limitations to this study. First, comorbidities were identified based on codes. Although many studies have reported that chronic anemia increases the likelihood of perioperative transfusion, we did not observe this^4,37^. However, anemia in this study was detected based on codes assigned within 1 year before the surgery, which may not accurately reflect the true number of patients with anemia. Second, certain variables were not available in the claims data, such as body weight, smoking history, and operative times. To minimize error, only unilateral reconstruction was included in the study since Big data analysis was used to estimate the clinical situation retrospectively, based on claims data. Although not included in this study, bilateral mastectomy is reported to increase the transfusion risk compared to unilateral mastectomy, regardless of the breast reconstruction method; therefore, additional caution is required when performing bilateral reconstruction^38^. Third, because this study was based on Big data using NHIS claims, it was difficult to assess the absolute diagnostic criteria for anemia and liver disease. Each medical institution or doctor likely applied their own clinical diagnostic criteria. Fourth, the transfusion timing was unknown, *i*.*e*., whether it was pre-, intra-, or postoperative. It is difficult to ascertain the chronological relationship between claims because the NHIS in Korea bills by merging all claims that occurred in the hospitalization episode. Last, because the NHIS has only covered breast reconstruction after total mastectomy since April 2015, partial or simple mastectomy was not included in our study. Furthermore, due to the limitations of claims data, it is impossible to know whether the breast size or volume affected the bleeding in mastectomy. We also did not analyze the difference in transfusion rates according to the type of breast cancer. The literature on transfusion in breast cancer surgery reports that the frequency of transfusion was greater in patients under 40 years of age, TNM stage IV, undergoing preoperative chemotherapy, and modified radical mastectomy^39^. Despite these limitations, this is the first study to identify the risk factors for the need for transfusion in patients undergoing ABR using a nationwide Korean database. The results of this study may serve as a basis for future research.

## Conclusions

This study investigated the blood transfusion rates of patients undergoing ABR. The results of this study can serve as a reference for analysis of future trends in blood transfusion in patients undergoing ABR. The transfusion rate in this study was higher than in previous studies from other countries. The risk factors for transfusion should be identified to develop evidence-based guidelines to reduce the transfusion rates in these patients.

## Supplementary Information

Supplementary Table S1.

## Data Availability

The data that support the findings of this study are available Big Data Hub of the Health Insurance Review and Assessment Service (HIRA), but restrictions apply to the availability of these data, which were used under license for the current study, and so are not publicly available. Data are however available from the authors upon reasonable request and with permission of Big Data Hub of the Health Insurance Review and Assessment Service (HIRA).
