# Supplementary material for: Big data analysis of the risk factors and rates of perioperative transfusion in immediate autologous breast reconstruction
Source: Sci Rep. 2022 Mar 29;12:5314. doi: 10.1038/s41598-022-09224-7 (PMC8964768; doi:10.1038/s41598-022-09224-7)
Supplement: Supplementary file 1 — Supplementary Table S1. [file 41598_2022_9224_MOESM1_ESM.docx]

| Supplementary Table S1. The Healthcare Common Procedure Coding System of the Health Insurance Review and Assessment Service | |
| --- | --- |
| **Procedure codes for total mastectomy** | |
| N7130 | Radical mastectomy without axillary lymph node dissection |
| N7135 | Radical mastectomy |
| N7138 | Total mastectomy with axillary lymph node dissection |
| N7139 | Total mastectomy without axillary lymph node dissection |
| **Procedure codes for autologous breast reconstruction** | |
| N7140 | Autologous - LD muscle - LD flap |
| N7141 | Autologous - LD muscle - muscle-sparing LD myocutaneous flap (thoracodorsal artery perforator flap) |
| N7142 | Autologous - LD muscle - extended LD myocutaneous flap |
| N7143 | Autologous - pedicled TRAM flap |
| N7144 | Autologous - bipedicled TRAM flap |
| N7145 | Autologous - transverse TRAM free flap |
| N7146 | Autologous - muscle sparing TRAM free flap |
| N7147 | Autologous - deep inferior epigastric artery perforator free flap |
| **Procedure codes for breast reconstruction with implant** | |
| N7148 | Implant based - expander insertion (2-stage breast reconstruction, first stage) |
| N7149 | Implant based - DTI (direct-to-implant) |
| N7150 | Implant based - expander to permanent breast implant (2-stage breast reconstruction, second stage) |
| N7151 | Breast capsulectomy (Breast capsulorrhaphy, capsulotomy, capsular flap) |
| **Procedure codes for breast reconstruction with implant** | |
| X2021 | Whole blood and red blood cells, 320ml |
| X2022 | Whole blood and red blood cells, 400ml |
